# Predicting no-show appointments in a pediatric hospital in Chile using machine learning

**DOI:** 10.1007/s10729-022-09626-z

**Published:** 2023-01-28

**Authors:** J. Dunstan, F. Villena, J.P. Hoyos, V. Riquelme, M. Royer, H. Ramírez, J. Peypouquet

**Affiliations:** 1 Center for Mathematical Modeling (CNRS IRL2807), University of Chile, Santiago, Chile; 2 Departamento de Ciencia de la Computación and Instituto de Matemática Computacional, Pontificia Universidad Católica de Chile, Santiago, Chile; 3 Escuela de pregrados-Dirección Académica - Vicerrectoría, Universidad Nacional de Colombia Sede De La Paz, La Paz, Colombia; 4 Dr. Luis Calvo Mackenna Hospital, Santiago, Chile; 5 Mathematical Engineering Department, University of Chile, Santiago, Chile; 6 Bernoulli Institute for Mathematics, Computer Science and Artificial Intelligence, Faculty of Science and EngineeringUniversity of Groningen, Groningen, The Netherlands

**Keywords:** No-show patients, Appointments and schedules, Machine learning, Medical informatics, Public health

## Abstract

The Chilean public health system serves 74% of the country’s population, and 19% of medical appointments are missed on average because of no-shows. The national goal is 15%, which coincides with the average no-show rate reported in the private healthcare system. Our case study, Doctor Luis Calvo Mackenna Hospital, is a public high-complexity pediatric hospital and teaching center in Santiago, Chile. Historically, it has had high no-show rates, up to 29% in certain medical specialties. Using machine learning algorithms to predict no-shows of pediatric patients in terms of demographic, social, and historical variables. To propose and evaluate metrics to assess these models, accounting for the cost-effective impact of possible intervention strategies to reduce no-shows. We analyze the relationship between a no-show and demographic, social, and historical variables, between 2015 and 2018, through the following traditional machine learning algorithms: Random Forest, Logistic Regression, Support Vector Machines, AdaBoost and algorithms to alleviate the problem of class imbalance, such as RUS Boost, Balanced Random Forest, Balanced Bagging and Easy Ensemble. These class imbalances arise from the relatively low number of no-shows to the total number of appointments. Instead of the default thresholds used by each method, we computed alternative ones via the minimization of a weighted average of type I and II errors based on cost-effectiveness criteria. 20.4% of the 395,963 appointments considered presented no-shows, with ophthalmology showing the highest rate among specialties at 29.1%. Patients in the most deprived socioeconomic group according to their insurance type and commune of residence and those in their *second infancy* had the highest no-show rate. The history of non-attendance is strongly related to future no-shows. An 8-week experimental design measured a decrease in no-shows of 10.3 percentage points when using our reminder strategy compared to a control group. Among the variables analyzed, those related to patients’ historical behavior, the reservation delay from the creation of the appointment, and variables that can be associated with the most disadvantaged socioeconomic group, are the most relevant to predict a no-show. Moreover, the introduction of new cost-effective metrics significantly impacts the validity of our prediction models. Using a prototype to call patients with the highest risk of no-shows resulted in a noticeable decrease in the overall no-show rate.

**Supplementary Information:**

The online version contains supplementary material available at 10.1007/s10729-022-09626-z.

## Highlights

- We predict the probability of patients missing their medical appointments, based on demographic, social and historical variables.
- For each day and specialty, we provide a short list with the appointments that are more likely to be missed. The length of the list is determined using cost-effectiveness criteria. The hospital management can then apply a reduced number of actions in order to prevent the no-show or mitigate its effect.
- The use of a prototype in the hospital resulted in an average of 10.3 percentage points reduction in no-shows when measured in an 8-week experimental design.

## Introduction

With a globally increasing population, efficient use of healthcare resources is a priority, especially in countries where those resources are scarce [21]. One avoidable source of inefficiency stems from patients missing their scheduled appointments, a phenomenon known as no-show [7], which produces noticeable wastes of human and material resources [17]. A systematic review of 105 studies found that Africa has the highest no-show (43%), followed by South America (28%), Asia (25%), North America (24%), Europe (19%), and Oceania (13%), with a global average of 23% [11]. In pediatric appointments, no-show rates range between 15% and 30% [11], and tend to increase with the patients’ age [33, 44].

To decrease the rate of avoidable no-shows, hospitals can focus their efforts in three main areas:

a) *Identifying the causes*. The most common one is forgetting the appointment, according to a survey in the United Kingdom [36]. Lacy et al. [26] identified three additional issues: emotional barriers (negative emotions about going to see the doctor were greater than the sensed benefit), perceived disrespect by the health care system, and lack of understanding of the scheduling system. In pediatric appointments, other reasons include caregiver’s issues, scheduling conflicts, forgetting, transportation, public health insurance, and financial constraints [11, 19, 23, 39, 44, 49].

b) *Predicting patients’ behaviour.* To this end, researchers have used diverse statistical methods, including logistic regression [5, 20, 22, 40], generalised additive models [43], multivariate [5], hybrid methods with Bayesian updating [1], Poisson regression [41], decision trees [12, 13], ensembles [14, 37], and stacking methods [46]. Their efficiency depends on the ability of predictors to compute the probability of no-show for a given patient and appointment. Among adults, the most likely to miss their appointments are younger patients, those with a history of no-show, and those from a lower socioeconomic background, but variables such as the time of the appointment are also relevant [11].

c) *Improving non-attendance rates using preventive measures.* A review of 26 articles from diverse backgrounds found that patients who received a text notification were 23% less likely to miss their appointment than those who did not [42]. Similar results were obtained for personal phone calls in adolescents [39]. Text messages have been observed to produce similar outcomes to telephone calls, at a lower cost, in both adults [10, 18] and pediatric patients [29].

In terms of implementing mitigation actions, overbooking can maintain an efficient use of resources, despite no-show [2, 25]. However, there is a trade-off between efficiency and service quality. For other strategies, see the work of Cameron et al. [6].

This work is concerned with prediction and prevention in a pediatric setting. This is particularly challenging as attendance involves patients and their caregivers, who can moreover change over time.

We use machine learning methods to estimate the probability of no-show in pediatric appointments, and identify which patients are likely to miss them. This prediction is meant to be used by the hospital to reduce no-show rates through personalised actions. Since public hospitals have scarce resources and a tight budget, we introduce new metrics to account for both the costs and the effectiveness of these actions, which marks a difference with the work presented by Srinivas and Salah [47], which considers standard machine learning metrics, and Berg et al. [2], which balances interventions and opportunity costs, among others.

The paper is organised as follows: Section 2 describes the data and our methodological approach. It contains data description, the machine learning methods, our cost-effectiveness metrics, and the deployment. Results are shown in Section 3, paying particular attention to the metrics we constructed to assess efficiency, and the impact of the use of this platform, measured in an experimental design. Section 4 contains our conclusions and gives directions for future research. Finally, some details concerning the threshold tuning, and the balance between type I and II errors are given in the Appendix.

## Materials and methods

### Data description

Dr. Luis Calvo Mackenna Hospital is a high-complexity pediatric hospital in Santiago. We analysed the schedule of medical appointments from 2015 to 2018, comprising 395,963 entries. It contains socioeconomic information about the patient (commune of residence, age, sex,^1^ health insurance), and the appointment (specialty, type of appointment, day of the week, month, hour of the day, reservation delay), as well as the status of the appointment (show/no-show).

**Fig. 1:**
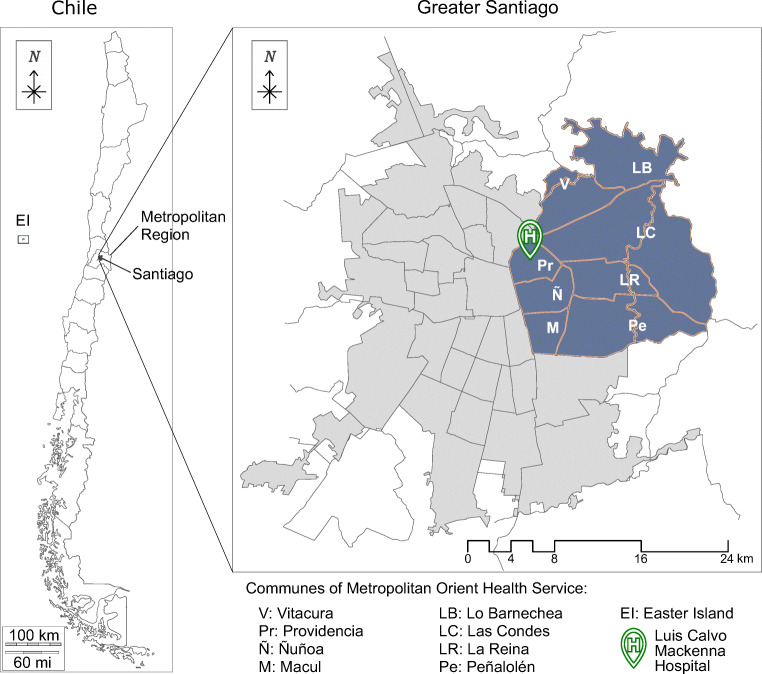
Map of communes that belong to the East Metropolitan Health Service

Although the hospital receives patients from the whole country, 70.7% of the appointments correspond to patients from the Eastern communes of Santiago (see Fig. 1). Among these communes, the poorest, Peñalolén, exhibits the highest percentage of no-show. Table 1 shows the percentage of appointments, no-shows and poverty depending on the patients’ commune of residence. For measuring poverty, we used the Chilean national survey Casen, which uses the multidimensional poverty concept to account for the multiple deprivations faced by poor people at the same time in areas such as education, health, among others [34].

**Table 1:** Location of the referred center, the proportion of patients from the total of appointments, no-show rate and proportion of the population in multidimensional poverty [34]

Referred from	Appts. %	No-show %	Poverty %
Peñalolén	31.1	23.8	26.3
Macul	12.4	23.5	13.5
Ñuñoa	8.9	21.9	5.8
Lo Barnechea	4.8	22.4	17.2
Las Condes	4.6	21.3	4.2
Providencia	4.1	20.2	3.4
La Reina	4.1	23.3	7.0
Vitacura	0.5	20.6	3.5
Easter Island	0.2	16.6	21.7
Other communes	11.1	16.7	−
Rest of the country	18.2	13.4	−

Since Dr. Luis Calvo Mackenna is a pediatric hospital, 99.2% of the appointments correspond to patients whose age at the day of the appointment is under 18 years. The distribution by age group is shown on Table 2.

**Table 2:** Appointments at Dr. Luis Calvo Mackenna displayed by age group

Life cycle grouping	Age Range	Percentage
Nursling	0-5 months	9.7%
First infancy	6 months-4 years	24.1%
Second infancy	5-11 years	39.2%
Teenagers	12-17 years	26.2%
Young adults	18-25 years	0.8%

Most appointments (96.5%) correspond to patients covered by the Public Health Fund FONASA. These patients are classified according to their socioeconomic status in groups A, B, C, and D. The income range for each group and the percentage of appointments at each level is shown in Table 3. During the time this study took place, patients in groups A and B had zero co-payment, while groups C and D had 10% and 20%, respectively. As of September 2022, due to new government policies, all patients covered in FONASA have a zero co-payment.

**Table 3:** Distribution of patients by grouping them according to socioeconomic status and type of appointment

Group	Description	Appointments %	No-Show %
Socioeconomic Status			
A	Without income/migrants	44.1	22.5
B	Less than US$425.	22.1	18.9
C	Between US$425 and US$620	13.0	18.9
D	Greater than US$621	17.3	18.3
Other	Without health insurance	2.0	20.4
Private	With private insurance	1.5	20.4
Type of appointment			
1st time appointment	First visit for a certain medical episode	23.1	24.1
Routine appointment	Medical controls that follow 1st appointments	63.7	18.6
1st time derived	Special slots derived from primary healthcare	8.7	26.8
Other	Mainly medical prescriptions	4.5	16.6

The type of appointment is also an important variable. Table 3 shows the percentage of appointments that correspond to first-time appointments, routine appointments, first-time appointments derived from primary healthcare, and others. The table shows each type’s volume and the percentage of no-shows for each type.

We analysed specialty consultation referrals both from within the hospital and from primary care providers. The dataset contains appointments from 25 specialties, which are shown in Table 4, along with the corresponding no-show rate. The no-show rate is uneven, and seems to be lower in specialties associated with chronic and life-threatening diseases (e.g. Oncology, Cardiology) than in other specialties (e.g. Dermatology, Ophthalmology).

**Table 4:** Medical and dental specialties in the dataset

Medical specialties (no-show %)	
Pulmonology (23.2)	Ophthalmology (30.3)
Cardiology (14.7)	Oncology (4.9)
General Surgery (16.9)	Otorhinolaryngology (22.7)
Plastic Surgery (14.2)	Psychiatry (24.0)
Dermatology (28.1)	Rheumatology (20.9)
Endocrinology (22.1)	Traumatology (19.9)
Gastroenterology (19.3)	Urology (19.3)
Gynecology (25.1)	Genetics (24.5)
Hematology (15.8)	Pediatrics (22.6)
Nephrology (18.4)	Infectology (23.7)
Neurology (28.3)	Parasitology (18.8)
Nutrition (27.6)	
Dental specialties (no-show %)	
Pediatric dentistry (24.9)	Orthodontics (18.4)

According to Dantas et al. [11], the patients’ no-show history can be helpful in predicting their behavior. In order to determine whether or not to use the complete history, we performed a correlation analysis between no-show and past non-attendance, as a function of the size of the look-back period. We observed that the Pearson correlation grows with the window size (0.09 at six months and 0.11 at 18 months), achieving a maximum correlation using the complete patient history (0.47). Note also that 20.3% of past appointments are missed when looking at time windows of only 12 months. This number grows to 55.2% when the window is 6 months. Due to the above reasons, we decided to consider all available no-show records.

The ultimate aim of this work is to identify which appointments are more likely to be missed. To do so, we developed models that classify patients based on attributes available to the hospital, which are described in Table 5.

**Table 5:** Description of the input features of the model

Feature name	Description	Type	Categories/range
Age	Age at the day of the appointment, as the position in the life cycle:	Categorical	Nursling (0-5 months), first infancy (6 months-4 years), second infancy (5-11 years), teenager (12-17 years), young adult (18-25 years)
Sex	Sex of the patient	Categorical	Male, female
Commune of residence	Location of residence of the patient at the commune level.	Categorical	Any of the 346 communes of Chile
Insurance	Insurance type	Categorical	Group A (person without housing or income, or migrant, Group B (monthly income < US $ 425), Group C (monthly income ∈ [US $ 425;US $621)), Group D (monthly income > US $ 621), Provisory Insurance (people without health insurance)
Day of the week	Day of the week of the appointment	Categorical	Monday - Friday
Month	Month of the appointment	Categorical	January - December
Hour of the day	Hour of the day of the appointment as a categorical feature	Categorical	8hrs - 17hrs (ranges of one hour)
Reservation delay	Time in weeks from the creation of the appointment generation and the appointment itself as a categorical feature.	Numerical	0,1,2,…
Historical no-show	Calculated as the no-show citations divided by total citations prior the current appointment.	Numerical	Number between 0 and 1
Historical no-show by specialty	Calculated as the no-show citations divided by total citations prior the current appointment, both with respect to the considered specialty.	Numerical	Number between 0 and 1
Type of appointment	Type of the appointment, regardless its medical specialty	Categorical	First-time appointment, routine appointment, and first-time appointment derived from primary healthcare (PHC)

### Machine learning methods

Our models predict the probability of no-show for a given appointment. This prediction problem was approached using supervised machine learning (ML) methods, where the *label* (variable to predict) was the appointment state: *show* or *no-show*. All the categorical features in Table 5 were transformed to one-hot encoded vectors. The numerical features (*historical no-show* and *reservation delay*) were scaled between 0 and 1.

In medical applications, the decisions and predictions of algorithms must be explained, in order to justify their reliability or trustworthiness [28]. Instead of deep learning, we preferred traditional machine learning, since its explanatory character [35] brings insight into the incidence of the variables over the output. This is particularly important because the hospital intends to implement tailored actions to reduce the no-show.

The tested algorithms, listed in Table 6, were implemented in Python programming language [50]. The distribution of the classes is highly unbalanced, with a ratio of 31:8 between show and no-show. To address the class imbalance we used algorithms suited for imbalanced learning implemented in imbalanced-learn [27] and scikit-learn [38]. To handle the problem of class balancing, RUSBoost [45] randomly under-samples the majority sample at each iteration of AdaBoost [16], which is a well-known boosting algorithm shown to improve the classification performance of weak classifiers. Similarly, the balanced Random Forest classifier balances the minority class by randomly under-sampling each bootstrap sample [8]. On the other hand, Balanced Bagging re-samples using random under-sampling, over-sampling, or SMOTE to balance each bootstrap sample [4, 32, 51]. The final classifier adapted to imbalanced data was Easy Ensemble, which performs random under-sampling. Then, it trains a learner for each subset of the majority class with all the minority training set to generate learner outputs combined for the final decision [30]. In turn, Support Vector Machine constructs a hyperplane to separate the data points into classes [9]. Logistic regression [15] is a generalized linear model, widely used to predict non-show [1, 7, 20, 22, 40]. We did not use stacking because these classifiers are likely to suffer from overfitting when the number of minority class examples is small [48, 52].

**Table 6:** Machine learning algorithms used in this work

imbalanced-learn	
RUS Boost	Balanced Random Forest
Balanced Bagging	Easy Ensemble
scikit-learn	
Logistic Regression	Random Forest
Ada Boost	Support Vector Machines

We trained and analyzed prediction models by specialty to ensure that each specialty receives unit-specific insights about the reasons correlated with their patients’ no-shows. Also, as shown in the Section 3, a single model incorporating specialty information through a series of indicator variables is less accurate than our specialty-based models.

The dataset was split by specialty, and each specialty subset was separated into training and testing subsets. The first subset was used to select optimal hyperparameters−selected via grid search on the values described in Table 7−and train machine learning algorithms. Due to computing power constraints, each hyperparameter combination performance was assessed using 3-fold cross-validation. The testing subset was used to obtain performance metrics.

**Table 7:** Hyperparameters for grid search

Model	Parameter	Values
AdaBoost	Decision tree max_depth	1, 2, 5, 8, 10, 15
	Decision tree min_samples_leaf	2, 3, 5, 10, 20, 40
	n_estimators	50, 100, 200, 300, 500, 750, 1000
	learning_rate	0.01, 0.05, 0.1, 0.2,None
Random Forest	bootstrap	True, False
Balanced Random	max_features	auto, sqrt
Forest (imblearn)	n_estimators	200, 400, 600, 800, 1000, 1200, 1400, 1600, 1800, 2000
	max_depth	10, 20, 30, 40, 50, 60, 70, 80, 90, 100,None
	min_samples_split	2, 5, 10, 50
Support Vector Machine	Kernel	linear, rbf
	C	1,10,100,1000
	Gamma (rbf kernel only)	1,0.1,0.001,0.0001
Logistic Regression	penalty	L1, L2
	C	0.00001, 0.0001, 0.001, 0.01, 0.1, 1, 10, 100, 1000
RUS Boost	n_estimators	50, 100, 400, 800, 1000, 1200, 1400, 1600, 1800, 2000
	replacement	True, False
Balanced Bagging	bootstrap	True, False
	bootstrap_features	True, False
	replacement	True, False
	n_estimators	10, 50, 100, 200, 500, 1000, 1200, 1400, 1600, 1800
EasyEnsemble	replacement	True, False
	n_estimators	10, 50, 100, 200, 500, 1000, 1200, 1400, 1600, 1800

The hyperparameters that maximised the metric given by (1-cost)⋆effectiveness (see Eq. 6 below) were used to train models using 10-fold cross-validation over the training subset to assess the best algorithm to use for specialty model training. Then, these combinations of best hyperparameters and algorithms were tuned to optimise their classification thresholds, as explained in the Appendix. The tuple (hyperparameter, algorithm, threshold) constitutes a *predictive model*. Then, the best predictive model for each medical specialty is chosen as the one that maximises cost/effectiveness (see Eq. 5 below). See Section 2.3 for more details

### Cost-effectiveness metrics

Custom metrics were developed to better understand the behavior of the trained models, and assess the efficiency of the system. These metrics balance the effectiveness of the predictions and the cost associated with possible prevention actions. This is particularly relevant in public institutions, which have strong budget limitations.

The use of custom cost-effectiveness metrics has two advantages. Firstly, they account for operational costs and constraints in the hospital’s appointment confirmation process, while standard machine learning metrics do not. For instance, the number of calls to be made or SMSs to be sent, the number of telephone operators, etc., all incur costs that the hospital must cover. Secondly, they offer an evident interpretation of the results since we establish a balance between the expected no-show reduction and the number of actions to be made. For instance, a statement such as “in order to reduce the no-show in ophthalmology by 30%, we need to contact 40% of daily appointments” can be easily understood by operators and decision-makers.

To construct these metrics, we used the proportion *P*_*C*_ of actions to be carried out, based on model predictions:

1 $$  P_C = \frac{ \text{FP} + \text{TP} }{N}, $$

where FP and TP are the number of false and true positives, respectively (analogously for FN and TN); and *N* = FP + TP + FN + TN is the total number of appointments (for the specialty). This quantity can be seen as a proxy of the cost of actions taken to prevent no-shows.

The second quantity used to define our custom metrics is the proportion *P*_*R*_ of no-show reduction, obtained from model predictions. First, let NSP_*i*_ be the existing no-show rate, and NSP_*f*_ be the no-show rate obtained after considering that all TP cases attend their appointment. That is:

2 $$ \begin{array}{@{}rcl@{}} \text{NSP}_i &=& \frac{ \text{FN} + \text{TP} }{N}, \end{array} $$

3 $$ \begin{array}{@{}rcl@{}} \text{NSP}_f &=& \frac{ \text{FN} }{ N }. \end{array} $$

Then, *P*_*R*_, computed as

4 $$ P_R = 1 - \frac{ \text{NSP}_f }{ \text{NSP}_i }{{=1-\frac{ \text{FN} }{ \text{FN}+\text{TP} }=\frac{ \text{TP} }{ \text{FN}+\text{TP} }}}, $$

measures the effectiveness of the prediction. To assess the trade-off between cost and effectiveness, we defined metrics:

5 $$ \begin{array}{@{}rcl@{}} m_{1}&:=&\text{effectiveness / cost} = \frac{P_{R}}{P_{C}}, \end{array} $$

6 $$ \begin{array}{@{}rcl@{}} m_{2}&:=&\text{effectiveness $\cdot$ (1 - cost)} = P_{R} \cdot (1 - P_{C}). \end{array} $$

Here, *P*_*R*_ is the proportion of correctly predicted no-shows from the total actual no-shows, a measure of efficiency. Conversely, *P*_*C*_ corresponds to the proportion of predicted no-shows from the total analyzed appointments, a measure of cost (number of interventions to be performed). Hence, *m*_1_ is the ratio between the proportion of no-shows avoided by the intervention and the proportion of interventions. In turn, *m*_2_ is the product (combined effect) of the proportion of no-shows avoided by intervention and the proportion of shows predicted (appointments no to be intervened).

Thus, an increase of a 10% in *m*_1_ can be produced by a 10% increase of *P*_*R*_ (an increase of correctly predicted no-shows) or a 10% decrease of *P*_*C*_ (decrease in the number of interventions to be performed). Similarly, an increase of a 10% of *m*_2_ can be produced by a 10% increase of *P*_*R*_ (an increase of correctly predicted no-shows) without performing more interventions, or a 10% increase of 1 − *P*_*C*_ (decrease in the number of interventions to be performed) without changing *P*_*R*_.

These two metrics are used to construct and select the best predictive models for each specialty. This decision is supported by the fact that, by construction, both metrics have higher values when the associated model performs better in a (simple) cost-effectiveness sense and is therefore preferred according to our methodology. Then, since the range of *m*_2_ is bounded (it takes values between 0 and 1), we used it as the objective function for hyperparameter optimization, which is an intermediate process to construct our predictive models. On the other hand, since *m*_1_ is slightly easier to interpret (but possibly unbounded), we used it to select the best predictive model for each studied medical specialty. An analysis of our classification metrics against Geometric Mean (GM) and Matthews’s Correlation Coefficient (MCC) is shown in the Appendix. This is carried out to analyze the bias of these two metrics in the context of an imbalanced dataset.

Regarding the limitations of the proposed metrics, we noticed that, in some occasional cases, the use of *m*_1_ recommended very few actions. Indeed, few medical appointments with high no-show probability generate a high classification threshold, yielding a high value of *m*_1_. For example, when the model recommends confirming the top 1% of the appointments (i.e., *P*_*C*_ = 0.01), but this also reduces the no-show rate by 5% (i.e., *P*_*R*_ = 0,05), we obtain a *m*_1_ = 5. To overcome this problem in a heuristic way, and also for practical reasons (values of *m*_2_ are bounded), we use metric *m*_2_ for the hyperparameters optimization process. However, we keep *m*_1_ to select the best predictive model for each specialty because it is easier to interpret than *m*_2_.

Another approach used in the literature is the comparision of models through costs instead of a cost-effectiveness analysis—for example, the minimization of both the costs of outreaches and the opportunity cost of no-shows. For instance, in the context of overbooking, Berg et al. [2] suggested that the cost function to be minimized could balance the cost of prevention (predicted no-shows multiplied by the cost of intervention) and the cost of no-shows (real no-shows multiplied by the cost of medical consultation). This approach could be adapted to our context to assess mitigation actions (such as phone calls) through more realistic criteria. However, this is beyond the scope of this research and will be the object of future studies.

### Deployment

We designed a computational platform to implement our predictive models as a web application. The front- and back-end were designed in Python using the Django web framework. The input is a spreadsheet containing the appointment’s features, such as patient ID and other personal information, medical specialty, date, and time. This data is processed to generate the features described in Table 5.

For each specialty, the labels of all appointments are predicted using the best predictive model. The appointments are sorted in descending order according to the predicted probability of no-show, along with the patient’s contact information. The hospital may then contact the patients with the highest probability of no-show to confirm the appointment.

## Results

Table 8 shows the best model for each specialty analyzed and provides the values for the *m*_1_ and *m*_2_ metrics, along with the Area Under the Receiver Operating Characteristics Curve (AUC) metric. Please check the Appendix (Table 15) for additional metrics corresponding to the best model in each specialty.

**Table 8:** Performance of the best model for each medical specialty

Specialty	Algorithm	Threshold	*P*_*C*_	*NSP*_*i*_	*NSP*_*f*_	*P*_*R*_	*m*_1_	*m*_2_	AUC
Cardiology	RandomForestClassifier	0.55	0.10	0.16	0.13	0.18	1.76	0.16	0.63
Dermatology	RandomForestClassifier	0.56	0.13	0.26	0.21	0.22	1.61	0.19	0.65
Endocrinology	RandomForestClassifier	0.54	0.20	0.21	0.14	0.33	1.68	0.27	0.66
Gastroenterology	BalancedBaggingClassifier	0.68	0.11	0.19	0.15	0.21	1.90	0.19	0.65
General surgery	LogisticRegression	0.67	0.19	0.13	0.08	0.40	2.17	0.33	0.72
Genetics	BalancedRandomForestClassifier	0.57	0.18	0.23	0.18	0.24	1.32	0.20	0.57
Gynecology	BalancedBaggingClassifier	0.65	0.14	0.24	0.19	0.22	1.54	0.19	0.61
Hematology	RandomForestClassifier	0.54	0.16	0.16	0.10	0.38	2.31	0.32	0.73
Infectology	RandomForestClassifier	0.57	0.11	0.26	0.21	0.21	1.79	0.18	0.64
Nephrology	BalancedBaggingClassifier	0.73	0.11	0.15	0.12	0.23	2.17	0.21	0.69
Neurology	BalancedBaggingClassifier	0.64	0.12	0.26	0.20	0.23	1.91	0.20	0.68
Nutrition	LogisticRegression	0.65	0.10	0.32	0.27	0.16	1.53	0.14	0.60
Oncology	RandomForestClassifier	0.50	0.09	0.04	0.03	0.29	3.26	0.26	0.72
Ophtalmology	BalancedRandomForestClassifier	0.65	0.13	0.31	0.24	0.21	1.61	0.18	0.62
Orthodontics	BalancedBaggingClassifier	0.63	0.17	0.21	0.11	0.47	2.87	0.40	0.80
Otorhinolaryngology	BalancedBaggingClassifier	0.61	0.18	0.22	0.14	0.37	2.07	0.30	0.69
Parasitology	BalancedBaggingClassifier	0.72	0.12	0.17	0.12	0.26	2.20	0.23	0.65
Pediatric dentistry	BalancedBaggingClassifier	0.67	0.11	0.30	0.24	0.20	1.86	0.18	0.66
Pediatrics	BalancedRandomForestClassifier	0.63	0.13	0.25	0.19	0.23	1.75	0.20	0.64
Plastic surgery	BalancedRandomForestClassifier	0.67	0.21	0.10	0.05	0.47	2.22	0.37	0.76
Psychiatry	RandomForestClassifier	0.56	0.14	0.25	0.19	0.25	1.78	0.21	0.65
Pulmonology	BalancedRandomForestClassifier	0.61	0.27	0.17	0.09	0.49	1.85	0.36	0.74
Rheumatology	BalancedRandomForestClassifier	0.66	0.11	0.22	0.18	0.16	1.54	0.14	0.60
Traumatology	BalancedBaggingClassifier	0.65	0.13	0.18	0.14	0.22	1.71	0.20	0.63
Urology	BalancedRandomForestClassifier	0.61	0.13	0.19	0.15	0.23	1.73	0.20	0.63

**Fig. 2:**
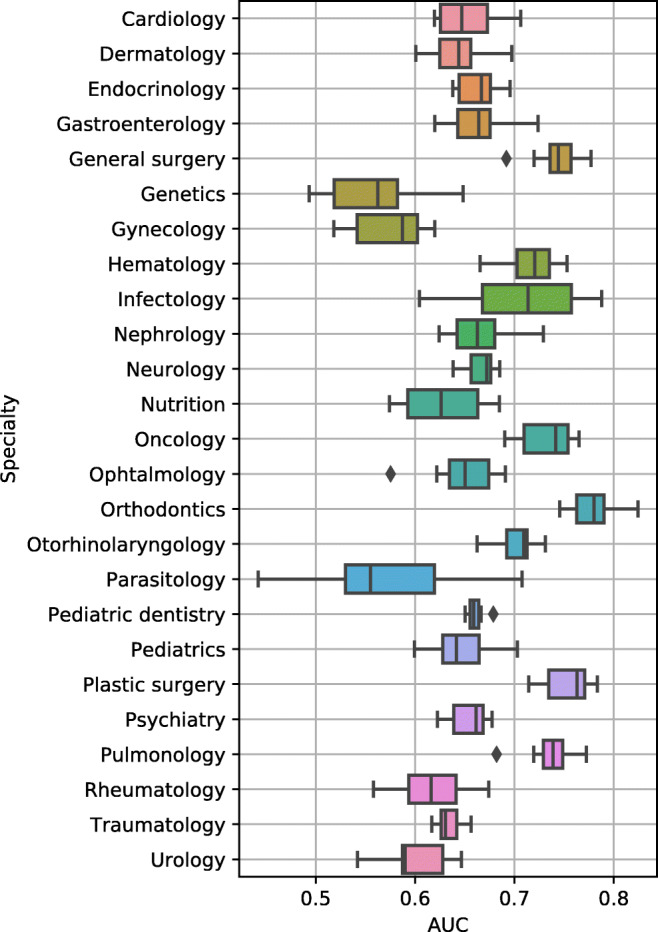
Cross-validated AUC performance of the best (hyperparameter, model) combination

Cross-validated AUC performance of the best (hyperparameter, model) combination with its deviations is also shown in Fig. 2. Our proposed metrics correlate with the AUC performance (0.78 and 0.89 Pearson correlation for *m*_1_ and *m*_2_, respectively), suggesting our custom-tailored metrics conform with the well-known AUC metric. However, in contrast to AUC, metrics *m*_1_ and *m*_2_ can be related to the trade-off between costs and effectiveness. Our proposed single-specialty models achieve a weighted *m*_1_ of 3.33 (0.83 AUC), in contrast to the single model architecture for all specialties that achieves an *m*_1_ of 2.18 (0.71 AUC). Balanced Random Forest and Balanced Bagging were the best classifiers in 8 and 9 specialties, respectively. The imbalanced-learn methods outperformed the scikit-learn ones in this study. Ensemble methods, such as BalancedBaggingClassifier, which combine multiple isolated models, usually achieve better results due to a lower generalization error. In addition, our dataset is imbalanced, so it is not surprising that the balanced versions of the classifiers are dominant. Interestingly, the three best algorithms (BalancedBaggingClassifier, Randomforestclassifier, and BalancedRandomForestClassifier) are based on bagging, which combines trees independently.

For each specialty, the results in Table 8 can be interpreted as follows: Suppose that there are 1,000 appointments and a historical no-show rate of 20%. Then, *P*_*C*_ = 0.27 means that our model recommends confirming the 270 appointments with the highest no-show probability. On the other hand, *P*_*R*_ = 0.49 means that this action may reduce the no-show rate from the original 20% to 10.2% (= (1-0.49) x 20%; see Eq. 4).

**Fig. 3:**
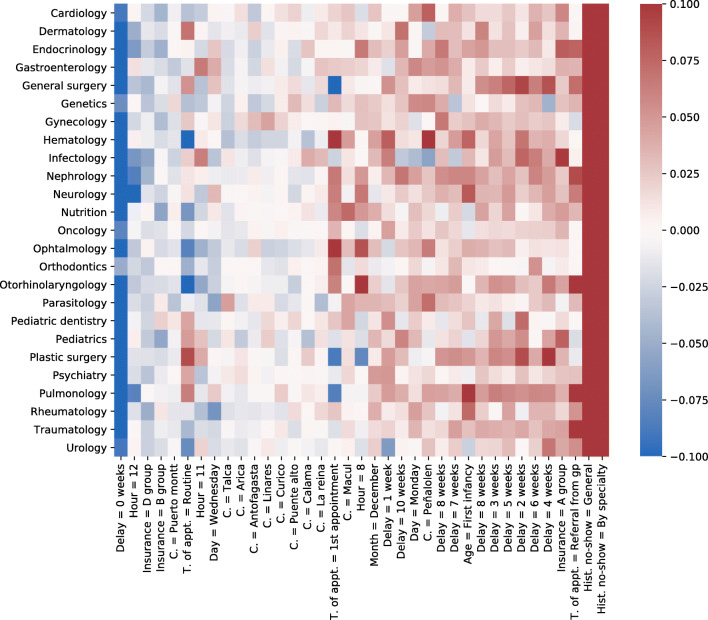
Features with the strongest label correlation by specialty. All correlations presented have p-values < 0.001

Table 9 and Fig. 3 show the features with the strongest correlation with no-show, overall and by specialty, respectively. The historical no-show and the reservation delay are the most correlated variables to no-show. A patient with a large historical no-show rate is likely to miss the appointment, and a patient whose appointment is scheduled for the ongoing week is likely to attend. First-time appointments are more likely to be missed. Patients are likely to miss an 8 am appointment, while they are more likely to attend at 11 am. These results are consistent with the analysis of a Chile dataset from 2012 to 2013 reported previously [24]. Peñalolén and Macul show a larger correlation with no-show. Patients belonging to Group A of the public health insurance (lowest income) are more likely not to attend, contrary to those in Group D (highest income). Interestingly, patients from outside Santiago are more likely to attend. Age, sex, and month of the appointment show a weaker correlation with no-show, which is consistent with the results obtained by Kong et al. [24].

**Table 9:** Correlations between no-show and features

Feature	Correlation
Historical no-show	0.16
Reservation delay = 0 weeks	− 0.15
Historical no-show by specialty	0.15
Appointment type = routine appointment	− 0.07
Commune of residence = outside Santiago	− 0.07
Hour = 8	0.06
Commune of residence = Peñalolén	0.05
Appointment type = 1st appointment	0.05
Appointment type = 1st appointment PHC	0.05
Insurance = A Group	0.04
Reservation delay = 5 weeks	0.03
Commune of residence = Macul	0.03
Day of the week = Monday	0.03
Reservation delay = 6 weeks	0.03
Commune of residence = others in Santiago	− 0.03
Reservation delay = 3 weeks	0.03
Insurance = D Group	− 0.03
Day of the week = Wednesday	− 0.03
Hour of the day = 11	− 0.02

All correlations had a p-value < 0.001

Correlation with no-shows is not always coherent with the prediction power of the features. Moreover, both may change from one specialty to another, which further justifies our decision to model no-shows by specialty. Table 10 displays the correlation with no-shows, while Table 11 shows the predictive power of features for pulmonology.

**Table 10:** Correlations between no-show and features: Pulmonology

Feature	Correlation
Reservation delay = 0 weeks	− 0.20
Historical no-show	0.15
Appointment type = 1st appointment	0.08
Appointment type = 1st appointment PHC	0.08
Reservation delay = 30-50 weeks	0.08
Age = first infancy	0.07
Hour = 15	0.05
Insurance = A Group	0.05
Commune of residence = Peñalolén	0.05
Age = second infancy	− 0.05
Month = May	− 0.04
Hour = 12	− 0.04
Month = December	0.03
Day of the week = Monday	0.01

All correlations had a p-value < 0.001

**Table 11:** Feature importance in pulmonology (Balanced Random Forest Classifier)

Feature	Importance
Reservation delay = 0 weeks	0.13
Historical no-show	0.09
Hour = 15	0.03
Day of the week = Tuesday	0.01
Commune of residence = Peñalolén	0.01
Day of the week = Thursday	0.01
Age = Nursling	0.01
Sex = male	0.01
Hour = 9	0.01
Appointment type = 1st appointment PHC	0.01

The information for the remaining specialties can be found in the Supplementary Material.

**Fig. 4:**
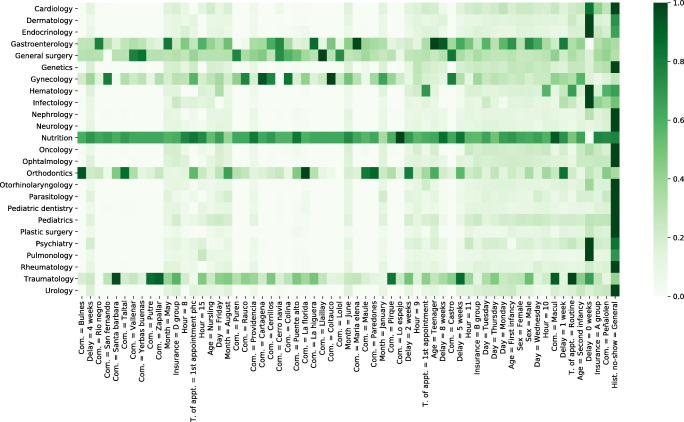
Features with the strongest Gini importance by specialty model

Figure 3 shows the features with the strongest label correlation for each specialty. Figure 4 presents a heatmap based on the seven most important features by specialty, in terms of their predictive power. To do so, the Gini or Mean Decrease Impurity [3] was sorted in descending order to their overall importance. In most specialties, no-show can be predicted by a small number of features, as shown by the sparsity of the corresponding lines. Some specialties−especially gastroenterology, general surgery, gynecology, nutrition, and traumatology−have a more complex dependence. Table 12 shows the features, calculated with the Gini importance, with the highest frequency. Historical no-show, Peñalolen commune, insurance group A and the minimal reservation delay appear consistently. Although there is a strong similarity between Tables 9 and 12, there are also differences. For example, *historical no-show by specialty* and *commune of residence outside Santiago* are strongly correlated with no-show, but their overall predictive importance is low.

**Table 12:** List of most recurring features

Feature	Count
Historical no-show	19
Insurance = A Group	16
Commune of residence = Peñalolen	16
Reservation delay = 0 weeks	15
Age = second infancy	9
Appointment type = routine appointment	6
Reservation delay = 1 weeks	6
Commune of residence = Macul	5
Hour = 10	5
Day of the week = Thursday	3
Hour = 11	3
Insurance = B Group	3
Day of the week = Tuesday	3
Sex = male	3
Day of the week = Monday	3
Age = first infancy	3
Sex = female	3
Day of the week = Wednesday	3
Appointment type = first appointment	2
Hour = 9	2

As shown in Table 8, the implementation of actions based on this model may yield a noticeable reduction of no-show (as high as 49% in pulmonology).

### Experimental design

The impact on no-shows of having appointments ordered by their risk of being missed was measured in collaboration with the hospital. We set an experimental design to measure the effect of phone calls made according to our models. This occurred between the 16th of November 2020 and the 15th of January 2021. The hospital does not receive patients on weekends, and we did not carry out follow-ups during the week between Christmas and new-year. Hence, we performed an 8-week experimental design in normal conditions.

On a daily basis, the appointments scheduled for the next working day were processed by our models to obtain an ordered list, sorted by no-show probability from highest to lowest. Then, the hospital’s call center reminded (only) the scheduled appointments classified as possible no-shows by our predictive models for the specialties selected for the experiment (see paragraph below). All of these appointments had been pre-scheduled in agreement with the patients. These reminders were performed before 10 AM.

We analyzed 4,617 appointments from four specialties: Dermatology, Neurology, Ophthalmology, and Traumatology. These specialties were chosen together with the hospital, due to their high appointment rates and significant no-show rates. Our predictive models recommended intervening in 495 appointments throughout the experimental design. That is, on average, approximately 10 appointments per day. From those appointments, 247 were randomly selected as a control group and 248 for the intervention group.

The no-show rates during these two months were 21.0% for the control group (which coincides with the historical NSP average of the hospital) and 10.7% for the intervention group, with a reduction of 10.3 percentage points (p-value$\sim 0.002$). Table 13 shows the no-show rates in both groups for the different specialties considered in the study.

**Table 13:** Comparison of no-show rates in control and intervention groups in experimental design

Specialty	No-show rate		Reduction in
	Control group	Intervention group	percentage points
Ophthalmology	29.6%	12.1%	17.5
Neurology	17.6%	5.0%	12.6
Traumatology	19.0%	10.3%	8.7
Dermatology	24.0%	21.1%	2.9
Total	21.0%	10.7%	10.3

To interpret these results in terms of metrics *m*_1_ and *m*_2_, first, we use the percentage of no-show of the control group as a proxy for the value NSP_*i*_. This percentage also coincides with the historical no-show of the hospital, which justifies this decision. We obtained *P*_*R*_ = (21.0*%* − 10.7*%*)/21.0*%* = 0.46 and *P*_*C*_ = 247/4,617 = 0.05. This can be read as follows: calling the top 5% of appointments ordered from higher to lowest no-show probability generates a 46% decrease in no-shows. Thus, in terms of the metrics, we get *m*_1_ = *P*_*R*_/*P*_*C*_ = 9.80 and *m*_2_ = *P*_*R*_(1 − *P*_*C*_) = 0.47.

## Conclusions, perspectives

We have presented the design and implementation of machine learning methods applied to the no-show problem in a pediatric hospital in Chile. It is the most extensive work using Chilean data, and among the few in pediatric settings. The novelty of our approach is fourfold:

1. The use extensive historical data to train machine learning models.
2. The most suitable machine learning model for each specialty was selected from various methods.
3. The development of tailored cost-effectiveness metrics to account for possible preventive interventions.
4. The realization of an experimental design to measure the effectiveness of our predictive models in real conditions

Our results show a notorious variability among specialties in terms of the predictive power of the features. Although reservation delay and historical no-show are consistently strong predictors across most specialties, variables such as the patient’s age, time of the day, or appointment type must not be overlooked.

Future work includes testing the effect of adding weather variables. However, including weather forecasts from external sources poses additional technical implementation challenges. Another interesting line of future research is measuring the predictive power of our methods for remote consultations using telemedicine. Finally, as said before, we use cost-effectiveness metrics to construct and select the best predictive models. These metrics are computed as the proportion of avoided no-shows and the proportion of appointments identified as possible no-shows. Although simple, these metrics were enough for our purposes. They permit us to consider the hospital’s needs where resources are scarce, and it is not desirable to contact many patients. However, considering other more complex cost metrics (such as in Berg et al. [2]) could bring realism to our methodology and can be the object of a future study.

Some of the limitations of this study are that we work in pediatric settings, and extending our work to adult appointments will require us to train the models again. We are currently working on that by gathering funding to study no-shows for adults and combining urban and rural populations. In addition, this paper shows only the reduction in no-shows that calling had compared to a control group. Future work could include cheaper forms of contacting patients, such as SMS or WhatsApp messages written by automatic agents.

The implementation of actions based on the results provided by our platform may yield a noticeable reduction of avoidable no-shows. Using a prototype at Dr. Luis Calvo Mackenna Hospital in a subset of medical specialties and a phone call intervention has resulted in 10.3 percentage points less no-show. This research is a concrete step towards reducing non-attendance in this healthcare provider. Other actions, such as reminders of the appointments via phone calls, text messages, or e-mail, special scheduling rules according to patient characteristics, or even arranging transportation for patients from far communes, could be implemented in the future. However, all these actions rely on a good detection of possible no-shows to maximize the effect subjected to a limited budget.

### Supplementary Information

ESM 1 (PDF 406 KB)

## Figures and Tables

**Fig. 5:**
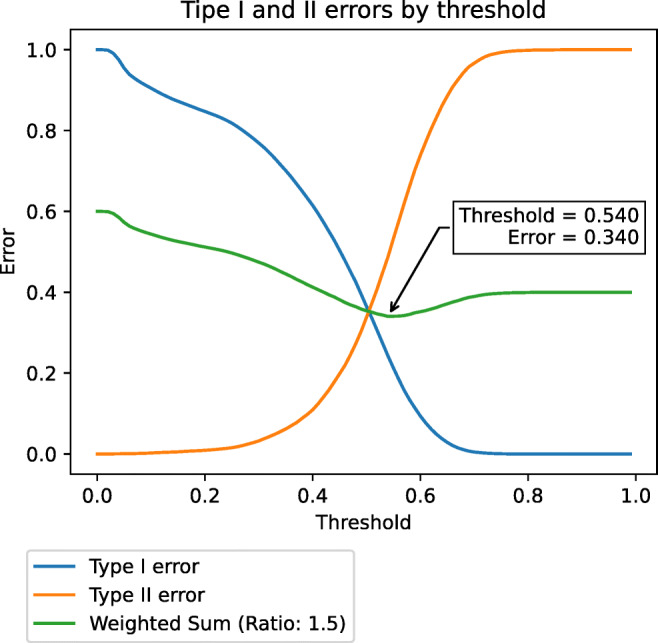
Type I and II errors as a function of the method classification threshold. Threshold *p* is selected as the minimiser of their weighted sum

**Fig. 6:**
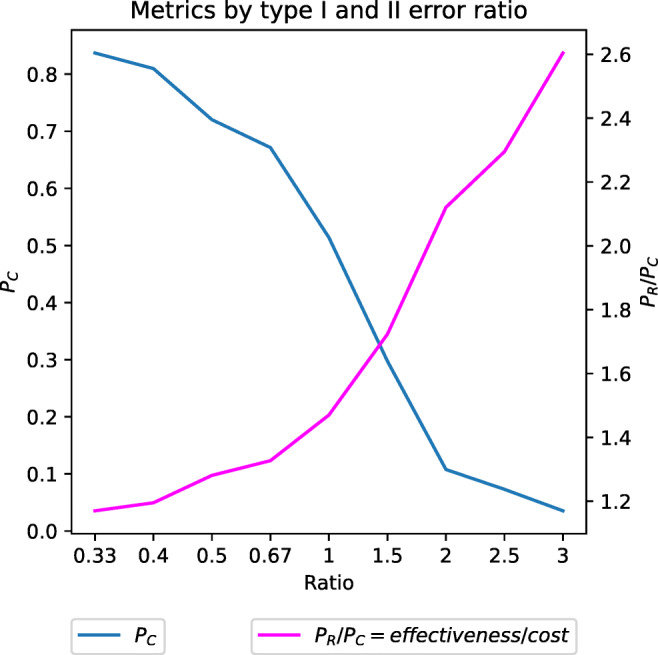
Performance metrics as a function of the type I and II weighting ratio

**Fig. 7:**
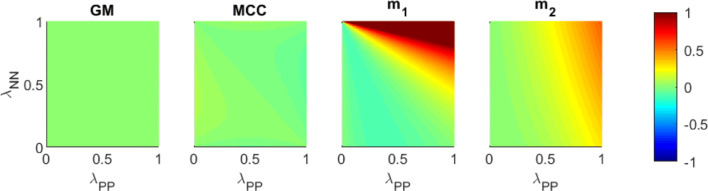
Heat maps of bias for each performance metrics with *δ* = 2 × 8/31 − 1

## Appendix A: Threshold tuning

The optimal classification thresholds were obtained by balancing type I and II errors (defined in Eqs. 7 and 8) for each method, following [22]. For the sake of completeness, we recall the mathematical relations involving these concepts:

7 $$  \mathrm{Type\ I\ error} = \frac{\text{FP}}{N - \text{NSP}_{i}}; $$

and

8 $$  \mathrm{Type\ II\ error} = \frac{\text{FN}}{\text{NSP}_{i}}. $$

where NSP_*i*_ is the existing no-show rate, FP and TP are the number of false positives and true positives, respectively (analogously for FN and TN); and *N* = FP + TP + FN + TN is the total number of appointments (for the analized specialty).

Instead of using the default thresholds, we computed the global minimum of a weighted sum of type I and II errors as shown in Fig. 5. More precisely, denote by *e*_1_(*p*) and *e*_2_(*p*) the type I and II errors as functions of the classification threshold *p* for each machine learning method, respectively, and let *w*_1_ and *w*_2_ be their respective weights. As explained in the next section, we considered the ratio *w*_1_/*w*_2_ = 1.5. Then, *p* is given by

9 $$ p\in \text{argmin} \{ w_{1} e_{1}(p)+w_{2}e_{2}(p)\}. $$

Once each method is trained, and its classification threshold tuned, we selected the best model (method, threshold) for each specialty based on the metrics described in Section 2.3.

### A.1 Ratio between type I and II errors

For the selection of weights *w*_1_ and *w*_2_ in problem (9), we analyzed the ratio *w*_1_/*w*_2_ between type I and II errors. For this, we computed *P*_*C*_ and *m*_1_ = *P*_*R*_/*P*_*C*_ as a function of *w*_1_/*w*_2_ (see Fig. 6). To write *P*_*C*_ and *P*_*R*_ in terms of FP, FN, TP, TN see Eqs. 1 and 4.

Huang and Hanauer [22] suggests that minimizing type I error is more critical than type II error in this context, suggesting a ratio higher than 1 (i.e., *w*_1_ > *w*_2_). We agree with this appreciation due to the limited resources in the public health sector and to ensure patient satisfaction. Figure 6 shows that, as the ratio increases, less patients will be acted upon, but our performance metric will also increase. Thus, by selecting a ratio higher than 1, we obtain a better cost-effectiveness. Although Fig. 6 corresponds only to an exercise for a given specialty and model, it is representative of the whole dataset. Based on the considerations above, we select a ratio of *w*_1_/*w*_2_ = 1.5, aiming at a greater patient satisfaction and a better cost-effectiveness.

### A.2 Metric bias

To analyze the performance of the metrics against feature imbalance, the measure designed by Luque et al. [31] was used. We determined the impact of the imbalance using the bias of the metric given by *B*_*μ*_(*λ*_*P**P*_,*λ*_*N**N*_,*δ*), where *λ*_*P**P*_ is the percent of true positive, *λ*_*N**N*_ is the percent of true negative, and *δ* the imbalance coefficient is given by 2*m*_*p*_/*m* − 1, where *m*_*p*_ is the total number of positive elements and *m* is the total number of elements.

Table 14 shows the definition of bias for the Geometric Mean (GM), Matthews’s Correlation Coefficient (MCC), and the proposed metrics *m*_1_ and *m*_2_. The first two were selected as benchmarks, since they are known to have a good performance with imbalanced datasets [31]. Since the imbalance coefficient *δ* of our dataset is 2 × 8/31 − 1, the bias depends only on *λ*_*P**P*_ and *λ*_*N**N*_. Figure 7 shows the bias in a heatmap. Metrics *m*_1_ and *m*_2_ have a low bias for most values of the parameters, with *m*_2_ showing the best performance. The use of both metrics allows to reduce the impact in areas with a high bias.

**Table 14:** Bias of performance metrics due to class imbalance

Metrics	Bias *B*_*μ*_(*λ*_*P**P*_,*λ*_*N**N*_,*δ*)
GM	0
MCC	$\frac {{\lambda _{PP}}+{\lambda _{NN}}-1}{2\sqrt {[{\lambda _{PP}}+(1-{\lambda _{NN}})\frac {1+\delta }{1-\delta }][{\lambda _{NN}}+(1-{\lambda _{PP}})\frac {1+\delta }{1-\delta }]}}- $
	$\frac {\lambda _{PP}+\lambda _{NN}-1}{2\sqrt {[\lambda _{PP}+(1-\lambda _{NN})][\lambda _{NN}+(1-\lambda _{PP})]}}$
*m*_1_	$\frac {2\lambda _{PP}}{\lambda _{PP}(1+\delta )+(1-\lambda _{NN})(1-\delta )} - \frac {2\lambda _{PP}}{\lambda _{PP}+(1-\lambda _{NN})}$
*m*_2_	$\frac {\lambda _{PP}}{2}(\lambda _{NN}(1-\delta )-\lambda _{PP}(1+\delta )) - \frac {\lambda _{PP}}{2}(\lambda _{NN}-\lambda _{PP})$

### A.3 ML metrics the best models for each specialty

Table 15 gives more information about the best model in each specialty.

**Table 15:** Additional performance metrics of the best model for each medical specialty

Specialty	Precision_*s**h**o**w*_	Precision_*n**o*−*s**h**o**w*_	Recall_*s**h**o**w*_	Recall_*n**o*−*s**h**o**w*_	*F*_1_ Score_*s**h**o**w*_	*F*_1_ Score_*n**o*−*s**h**o**w*_
Cardiology	0.85	0.29	0.91	0.18	0.88	0.22
Dermatology	0.76	0.42	0.90	0.22	0.82	0.28
Endocrinology	0.83	0.35	0.84	0.33	0.83	0.34
Gastroenterology	0.83	0.36	0.91	0.21	0.87	0.27
General surgery	0.91	0.28	0.85	0.40	0.88	0.33
Genetics	0.78	0.31	0.84	0.24	0.81	0.27
Gynecology	0.78	0.37	0.88	0.22	0.83	0.28
Hematology	0.88	0.37	0.88	0.38	0.88	0.37
Infectology	0.76	0.47	0.92	0.21	0.83	0.29
Nephrology	0.87	0.33	0.92	0.23	0.89	0.27
Neurology	0.77	0.50	0.92	0.23	0.84	0.31
Nutrition	0.70	0.49	0.92	0.16	0.80	0.24
Oncology	0.97	0.12	0.92	0.29	0.94	0.17
Ophtalmology	0.72	0.49	0.91	0.21	0.80	0.29
Orthodontics	0.87	0.59	0.92	0.47	0.89	0.53
Otorhinolaryngology	0.83	0.45	0.88	0.37	0.85	0.40
Parasitology	0.86	0.37	0.91	0.26	0.89	0.30
Pediatric dentistry	0.73	0.55	0.93	0.20	0.82	0.30
Pediatrics	0.78	0.43	0.90	0.23	0.84	0.30
Plastic surgery	0.93	0.22	0.81	0.47	0.87	0.30
Psychiatry	0.78	0.45	0.90	0.25	0.84	0.32
Pulmonology	0.88	0.32	0.78	0.49	0.83	0.39
Rheumatology	0.80	0.33	0.91	0.16	0.85	0.22
Traumatology	0.84	0.31	0.89	0.22	0.86	0.26
Urology	0.83	0.33	0.89	0.23	0.86	0.27

## Footnotes

1 Of the 395,963 appointments, there are 15 from intersex patients and 25 in which sex was marked as undefined. These appointments were not considered to create the model because small group sizes could cause model overfitting.
