# Supplementary material for: Predicting no-show appointments in a pediatric hospital in Chile using machine learning
Source: Health Care Manag Sci. 2023 Jan 28;26(2):313–29. doi: 10.1007/s10729-022-09626-z (PMC10257628; doi:10.1007/s10729-022-09626-z)
Supplement: Supplementary file 1 — (PDF 406 KB) [file 10729_2022_9626_MOESM1_ESM.pdf]

Supplementary Material

This section contains the ten (10) most Gini important features for each model that was selected for each specialty.

Table 1: Features of RandomForestClassifier: Cardiology

Feature	Value
Historical no-show = General	0.08
Reservation delay = 0 weeks	0.07
Insurance = A group	0.03
Day of the week = Monday	0.02
Commune of residence = Peñalolen	0.02
Commune of residence = Macul	0.02
Age = Second infancy	0.02
Sex = Female	0.02
Sex = Male	0.02
Insurance = B group	0.02

Table 2: Features of RandomForestClassifier: Dermatology

Feature	Value
Reservation delay = 0 weeks	0.11
Historical no-show = General	0.08
Type of appointment = Routine	0.03
Insurance = A group	0.02
Age = Second infancy	0.02
Sex = Female	0.02
Sex = Male	0.02
Commune of residence = Peñalolen	0.02
Reservation delay = 8 weeks	0.02
Day of the week = Wednesday	0.02

Table 3: Features of RandomForestClassifier: Endocrinology

Feature	Value
Reservation delay = 0 weeks	0.11
Historical no-show = General	0.07
Insurance = A group	0.03
Commune of residence = Peñalolen	0.02
Hour = 8	0.02
Type of appointment = Routine	0.02
Age = Second infancy	0.02
Insurance = B group	0.02
Type of appointment = 1st appointment	0.02
Day of the week = Tuesday	0.02

Table 4: Features of BalancedBaggingClassifier: Gastroenterology

Feature	Value
Age = Teenager	0.01
Commune of residence = Maria elena	0.01
Reservation delay = 8 weeks	0.01
Commune of residence = La higuera	0.01
Month = May	0.01
Reservation delay = 1 week	0.01
Commune of residence = Rio negro	0.01
Commune of residence = Sagrada familia	0.01
Commune of residence = Cerro navia	0.01
Sex = Male	0.01

Table 5: Features of LogisticRegression: General Surgery

Feature	Value
Commune of residence = Llaillay	6.79
Commune of residence = Yerbas buenas	5.27
Commune of residence = Castro	5.05
Commune of residence = Vallenar	4.69
Commune of residence = Puren	4.56
Commune of residence = Lolol	4.22
Commune of residence = Cerro navia	4.22
Commune of residence = Caldera	4.06
Commune of residence = Coelemu	3.90
Commune of residence = Calera de tango	3.52

Table 6: Features of BalancedRandomForestClassifier: Genetics

Feature	Value
Historical no-show = General	0.08
Commune of residence = Peñalolen	0.02
Insurance = A group	0.02
Hour = 9	0.02
Age = First infancy	0.02
Sex = Male	0.02
Age = Second infancy	0.02
Type of appointment = Routine	0.02
Hour = 10	0.02
Sex = Female	0.02

Table 7: Features of BalancedBaggingClassifier: Gynecology

Feature	Value
Commune of residence = Coltauco	0.009
Commune of residence = Cartagena	0.008
Commune of residence = Colina	0.007
Commune of residence = San fernando	0.007
Commune of residence = Castro	0.007
Commune of residence = Cerrillos	0.007
Commune of residence = Rauco	0.007
Commune of residence = Estacion central	0.006
Commune of residence = Lanco	0.006
Commune of residence = Valparaiso	0.006

Table 8: Features of RandomForestClassifier: Hematology

Feature	Value
Reservation delay = 0 weeks	0.09
Type of appointment = 1st appointment	0.06
Type of appointment = Routine	0.06
Historical no-show = General	0.06
Commune of residence = Peñalolen	0.05
Hour = 10	0.05
Day of the week = Friday	0.03
Reservation delay = 1 week	0.02
Hour = 9	0.02
Insurance = A group	0.02

Table 9: Features of RandomForestClassifier: Infectology

Feature	Value
Reservation delay = 0 weeks	0.12
Historical no-show = General	0.06
Insurance = A group	0.05
Commune of residence = Peñalolen	0.04
Reservation delay = 1 week	0.04
Type of appointment = Routine	0.03
Insurance = D group	0.03
Day of the week = Tuesday	0.03
Month = September	0.03
Commune of residence = Ñuñoa	0.02

Table 10: Features of BalancedBaggingClassifier: Nephrology

Feature	Value
Historical no-show = General	0.12
Reservation delay = 0 weeks	0.04
Commune of residence = Peñalolen	0.02
Insurance = A group	0.02
Hour = 10	0.02
Month = June	0.02
Hour = 11	0.02
Day of the week = Tuesday	0.02
Day of the week = Thursday	0.02
Commune of residence = Ñuñoa	0.01

Table 11: Features of BalancedBaggingClassifier: Neurology

Feature	Value
Historical no-show = General	0.13
Commune of residence = Peñalolen	0.02
Day of the week = Tuesday	0.02
Day of the week = Thursday	0.02
Age = Second infancy	0.02
Commune of residence = Macul	0.02
Insurance = A group	0.02
Age = First infancy	0.02
Hour = 10	0.02
Day of the week = Friday	0.02

Table 12: Features of LogisticRegression: Nutrition

Feature	Value
Commune of residence = Lo espejo	0.53
Commune of residence = Macul	0.44
Reservation delay = 8 weeks	0.33
Reservation delay = 5 weeks	0.29
Type of appointment = 1st appointment phc	0.28
Commune of residence = Puente alto	0.28
Commune of residence = Providencia	0.27
Month = November	0.26
Reservation delay = 7 weeks	0.25
Commune of residence = San javier	0.22

Table 13: Features of RandomForestClassifier: Oncology

Feature	Value
Historical no-show = General	0.08
Reservation delay = 0 weeks	0.05
Hour = 10	0.02
Insurance = A group	0.02
Hour = 11	0.02
Reservation delay = 1 week	0.02
Day of the week = Thursday	0.02
Sex = Male	0.02
Day of the week = Tuesday	0.02
Age = Second infancy	0.02

Table 14: Features of BalancedRandomForestClassifier: Ophthalmology

Feature	Value
Historical no-show = General	0.10
Insurance = A group	0.02
Commune of residence = Peñalolen	0.02
Reservation delay = 0 weeks	0.02
Age = Second infancy	0.02
Age = First infancy	0.02
Sex = Female	0.02
Sex = Male	0.02
Insurance = B group	0.02
Day of the week = Monday	0.02

Table 15: Features of BalancedBaggingClassifier: Orthodontics

Feature	Value
Commune of residence = La florida	0.008
Commune of residence = Bulnes	0.008
Commune of residence = Paredones	0.007
Commune of residence = Taltal	0.007
Reservation delay = 1 week	0.007
Commune of residence = Maule	0.007
Reservation delay = 2 weeks	0.007
Commune of residence = Isla de pascua	0.007
Commune of residence = San rosendo	0.007
Hour = 13	0.006

Table 16: Features of BalancedBaggingClassifier: Otorhinolaryngology

Feature	Value
Historical no-show = General	0.14
Reservation delay = 0 weeks	0.05
Type of appointment = 1st appointment	0.03
Commune of residence = Peñalolen	0.02
Insurance = A group	0.02
Reservation delay = 1 week	0.02
Day of the week = Monday	0.02
Age = Second infancy	0.02
Age = First infancy	0.02
Day of the week = Thursday	0.02

Table 17: Features of BalancedBaggingClassifier: Parasitology

Feature	Value
Historical no-show = General	0.13
Commune of residence = Peñalolen	0.03
Month = January	0.03
Day of the week = Wednesday	0.03
Month = August	0.03
Age = Second infancy	0.02
Reservation delay = 0 weeks	0.02
Month = April	0.02
Day of the week = Tuesday	0.02
Reservation delay = 4 weeks	0.02

Table 18: Features of BalancedBaggingClassifier: Pediatric Dentistry

Feature	Value
Historical no-show = General	0.14
Commune of residence = Peñalolen	0.02
Insurance = A group	0.02
Hour = 9	0.02
Hour = 10	0.02
Day of the week = Tuesday	0.02
Insurance = B group	0.02
Hour = 11	0.02
Month = August	0.02
Day of the week = Thursday	0.02

Table 19: Features of BalancedRandomForestClassifier: Pediatrics

Feature	Value
Historical no-show = General	0.09
Reservation delay = 0 weeks	0.03
Commune of residence = Peñalolen	0.02
Insurance = A group	0.02
Day of the week = Wednesday	0.02
Sex = Female	0.02
Insurance = B group	0.02
Sex = Male	0.02
Day of the week = Friday	0.02
Hour = 10	0.02

Table 20: Features of BalancedRandomForestClassifier: Plastic Surgery

Feature	Value
Historical no-show = General	0.14
Reservation delay = 0 weeks	0.04
Insurance = A group	0.02
Age = Teenager	0.02
Commune of residence = Peñalolen	0.02
Hour = 10	0.02
Reservation delay = 1 week	0.02
Age = First infancy	0.02
Type of appointment = Routine	0.02
Age = Second infancy	0.02

Table 21: Features of RandomForestClassifier: Psychiatry

Feature	Value
Reservation delay = 0 weeks	0.09
Historical no-show = General	0.08
Type of appointment = Routine	0.02
Hour = 11	0.02
Reservation delay = 1 week	0.02
Insurance = A group	0.02
Reservation delay = 4 weeks	0.02
Day of the week = Thursday	0.02
Day of the week = Tuesday	0.02
Commune of residence = Peñalolen	0.02

Table 22: Features of BalancedRandomForestClassifier: Pulmonology

Feature	Value
Reservation delay = 0 weeks	0.13
Historical no-show = General	0.09
Hour = 15	0.03
Day of the week = Tuesday	0.02
Commune of residence = Peñalolen	0.01
Day of the week = Thursday	0.01
Age = Nursling	0.01
Sex = Male	0.01
Hour = 9	0.01
Type of appointment = 1st appointment phc	0.01

Table 23: Features of BalancedRandomForestClassifier: Rheumatology

Feature	Value
Historical no-show = General	0.12
Reservation delay = 0 weeks	0.03
Day of the week = Wednesday	0.02
Commune of residence = Peñalolen	0.02
Insurance = B group	0.02
Commune of residence = Macul	0.02
Insurance = A group	0.02
Day of the week = Tuesday	0.02
Month = May	0.02
Hour = 10	0.02

Table 24: Features of BalancedBaggingClassifier: Traumatology

Feature	Value
Type of appointment = Routine	0.01
Commune of residence = Santa barbara	0.01
Commune of residence = Macul	0.01
Commune of residence = Zapallar	0.01
Reservation delay = 5 weeks	0.01
Commune of residence = Putre	0.01
Commune of residence = Pirque	0.01
Commune of residence = Negrete	0.01
Commune of residence = Los angeles	0.01
Commune of residence = Lebu	0.01

Table 25: Features of BalancedRandomForestClassifier: Urology

Feature	Value
Historical no-show = General	0.11
Commune of residence = Peñalolen	0.03
Age = Second infancy	0.03
Age = First infancy	0.03
Insurance = A group	0.02
Day of the week = Monday	0.02
Day of the week = Thursday	0.02
Day of the week = Tuesday	0.02
Hour = 12	0.02
Commune of residence = Macul	0.02

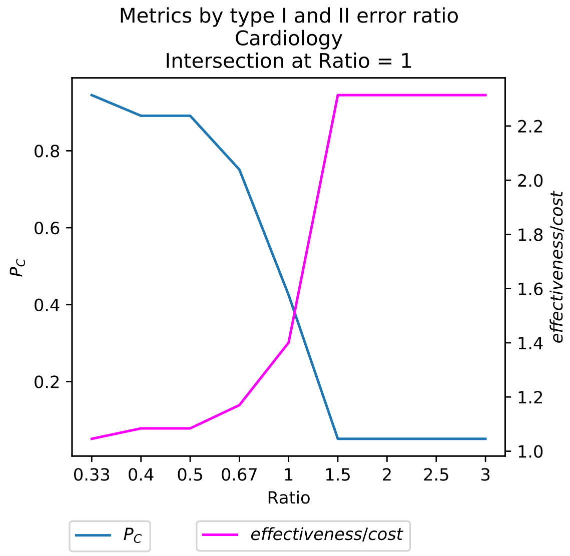

Figure 1: Performance metrics as a function of the type I and II weighting ratio in Cardiology.

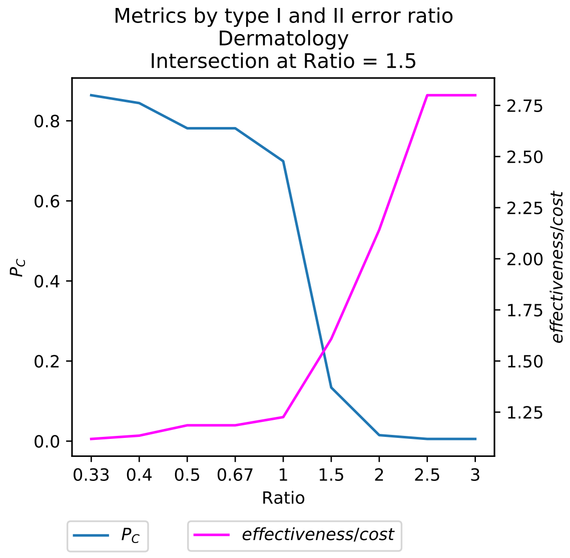

Figure 2: Performance metrics as a function of the type I and II weighting ratio in Dermatology.

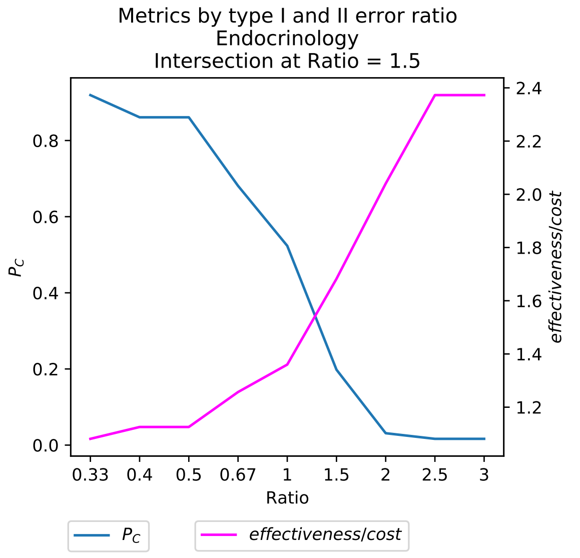

Figure 3: Performance metrics as a function of the type I and II weighting ratio in Endocrinology.

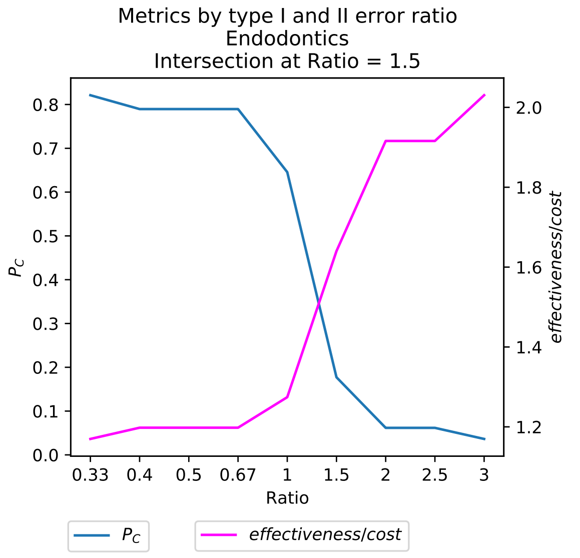

Figure 4: Performance metrics as a function of the type I and II weighting ratio in Endodontics.

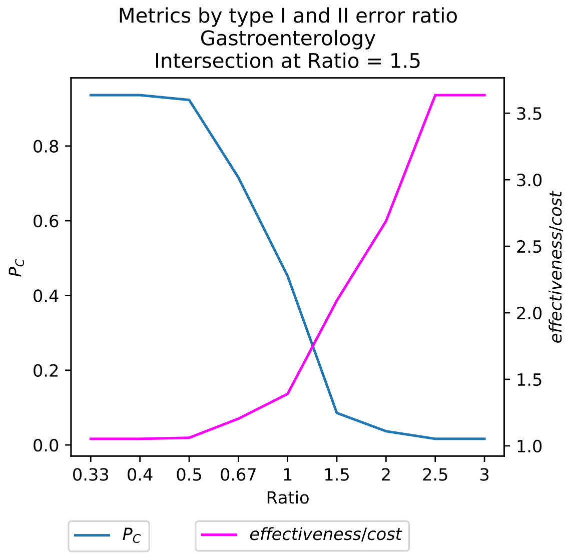

Figure 5: Performance metrics as a function of the type I and II weighting ratio in Gastroenterology.

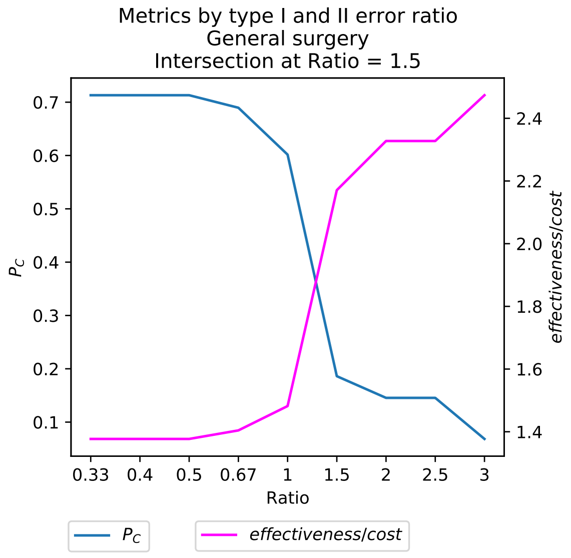

Figure 6: Performance metrics as a function of the type I and II weighting ratio in General surgery.

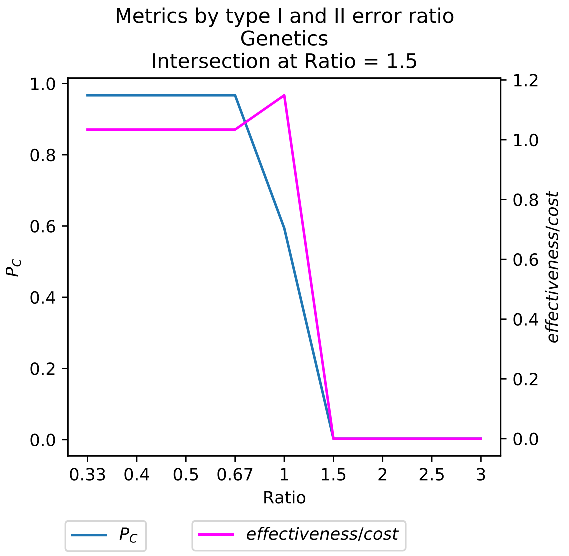

Figure 7: Performance metrics as a function of the type I and II weighting ratio in Genetics.

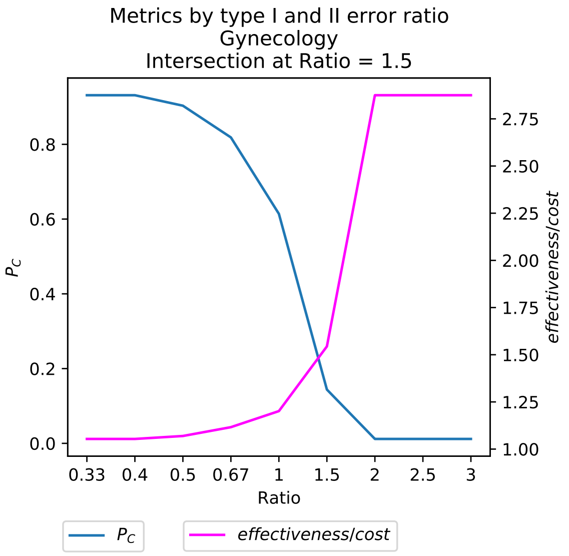

Figure 8: Performance metrics as a function of the type I and II weighting ratio in Gynecology.

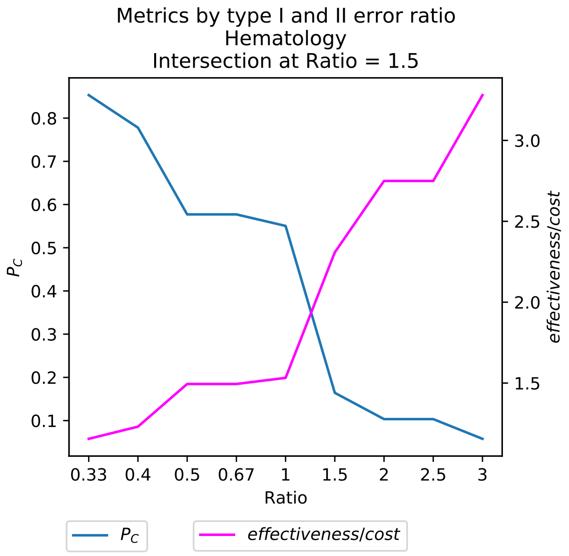

Figure 9: Performance metrics as a function of the type I and II weighting ratio in Hematology.

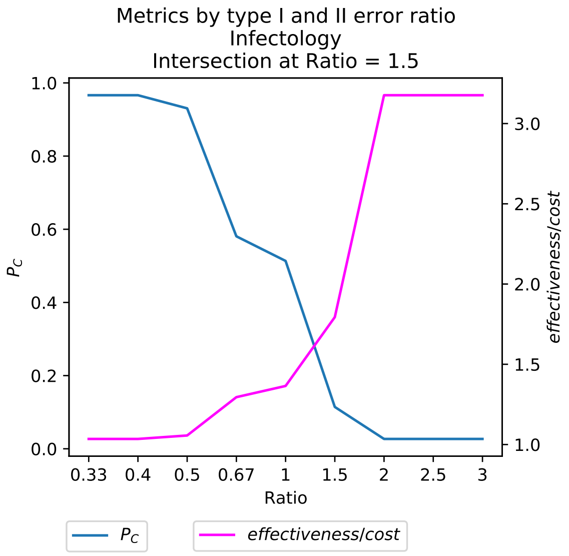

Figure 10: Performance metrics as a function of the type I and II weighting ratio in Infectology.

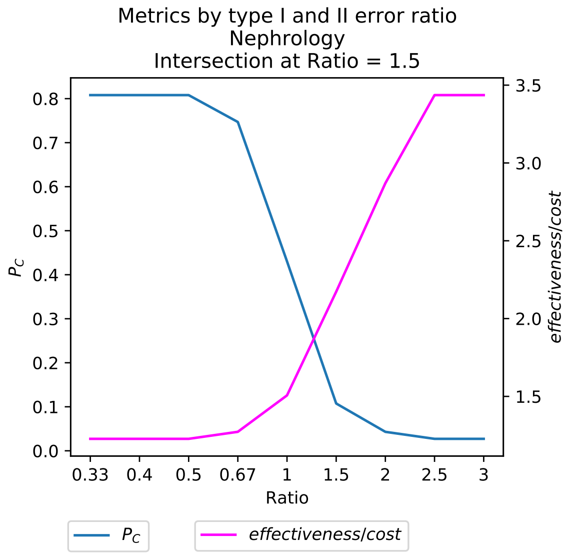

Figure 11: Performance metrics as a function of the type I and II weighting ratio in Nephrology.

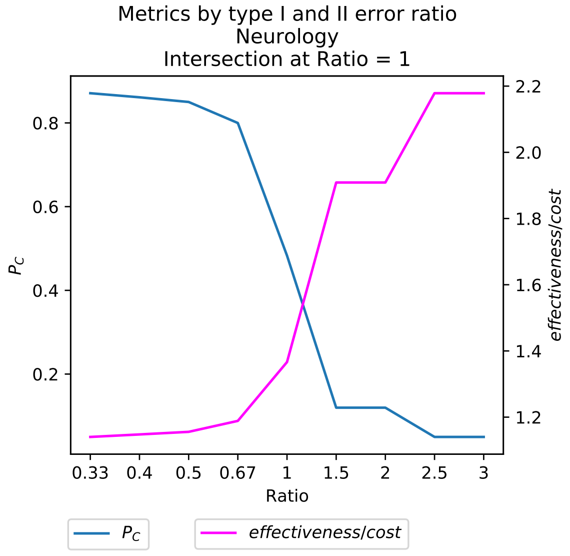

Figure 12: Performance metrics as a function of the type I and II weighting ratio in Neurology.

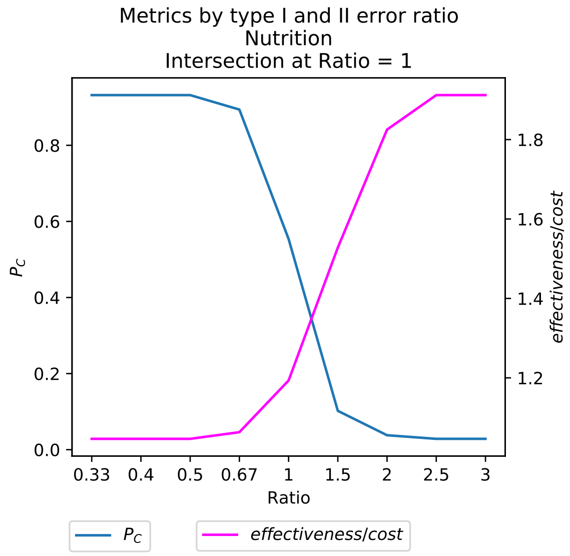

Figure 13: Performance metrics as a function of the type I and II weighting ratio in Nutrition.

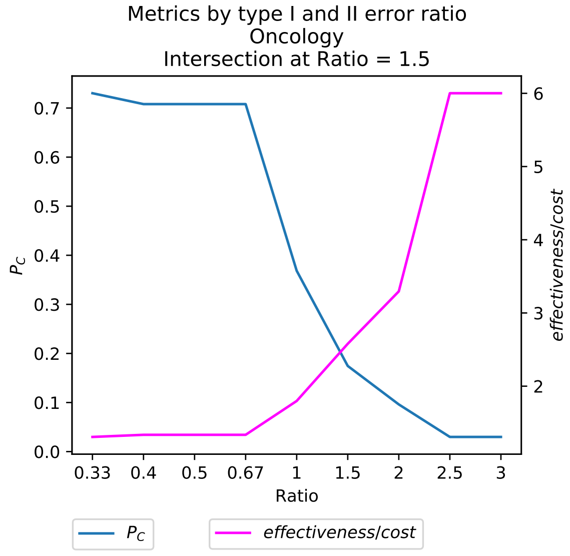

Figure 14: Performance metrics as a function of the type I and II weighting ratio in Oncology.

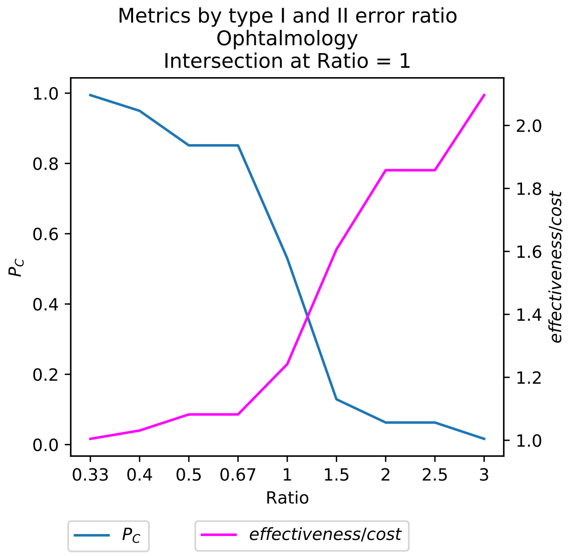

Figure 15: Performance metrics as a function of the type I and II weighting ratio in Ophtalmology.

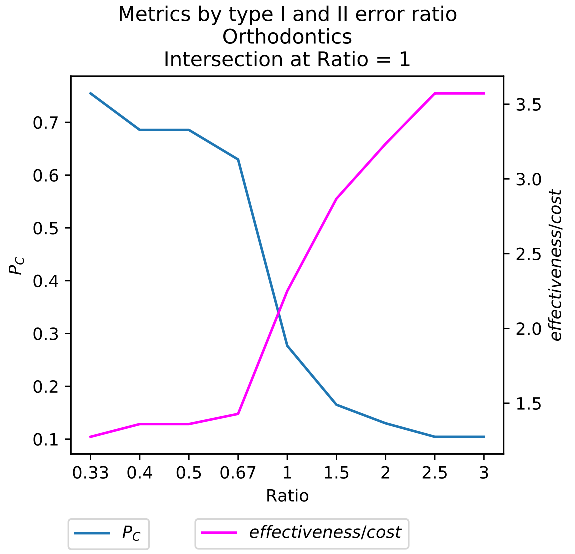

Figure 16: Performance metrics as a function of the type I and II weighting ratio in Orthodontics.

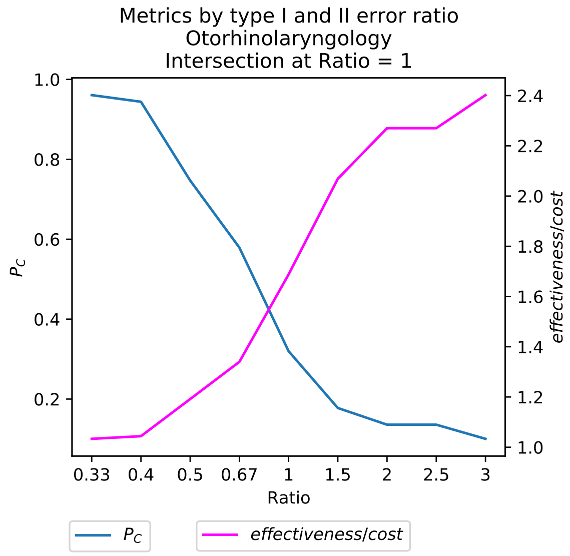

Figure 17: Performance metrics as a function of the type I and II weighting ratio in Otorhinolaryngology.

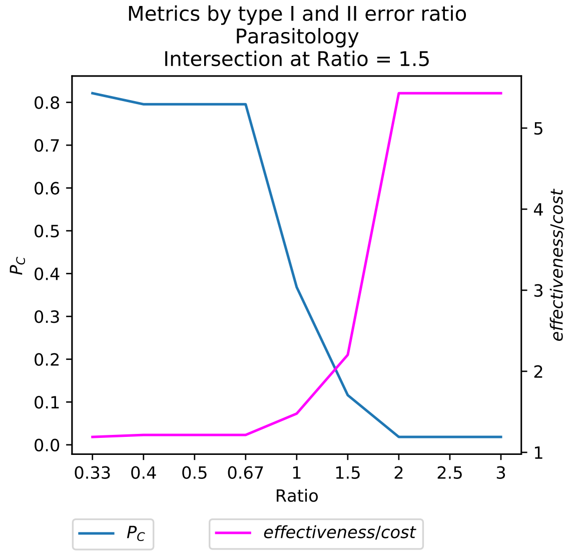

Figure 18: Performance metrics as a function of the type I and II weighting ratio in Parasitology.

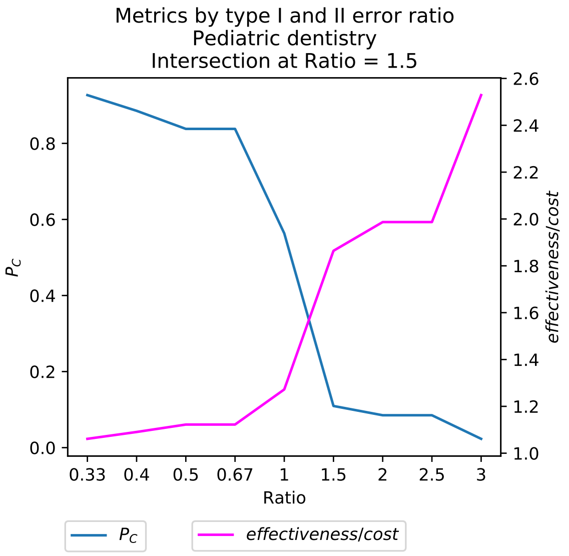

Figure 19: Performance metrics as a function of the type I and II weighting ratio in Pediatric dentistry.

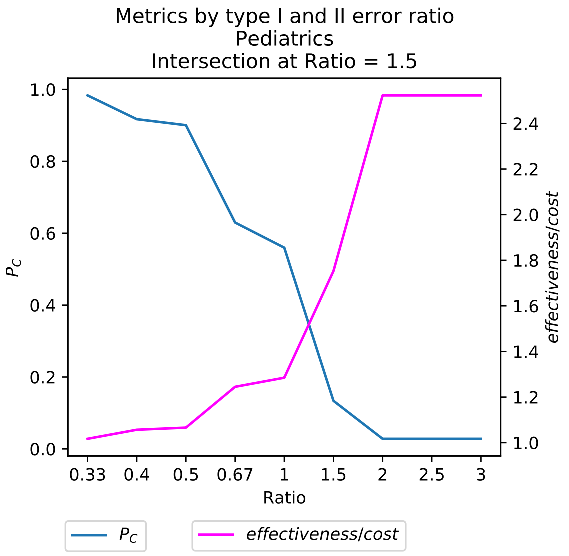

Figure 20: Performance metrics as a function of the type I and II weighting ratio in Pediatrics.

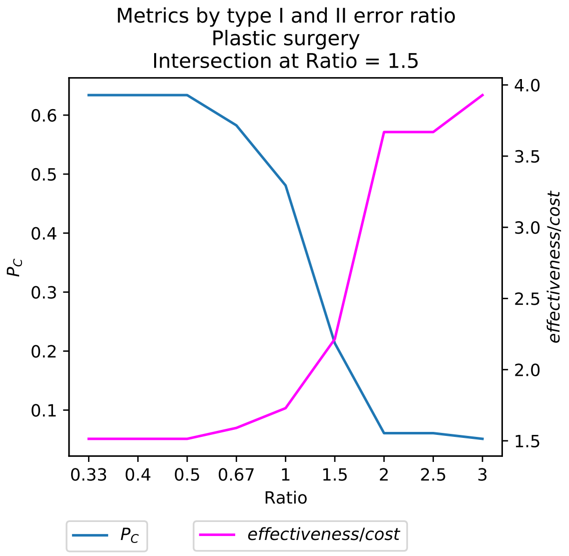

Figure 21: Performance metrics as a function of the type I and II weighting ratio in Plastic surgery.

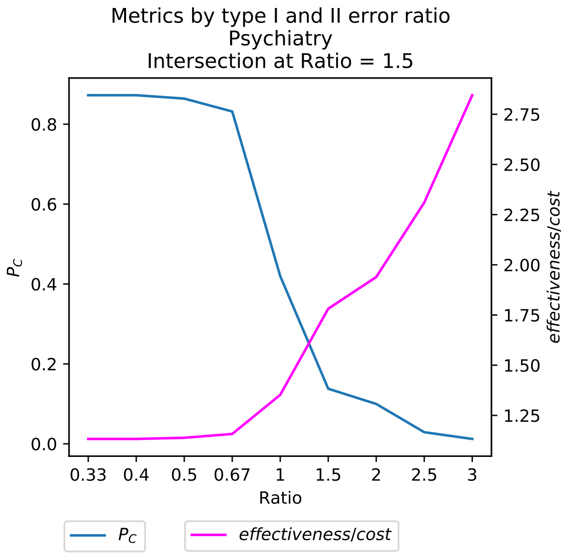

Figure 22: Performance metrics as a function of the type I and II weighting ratio in Psychiatry.

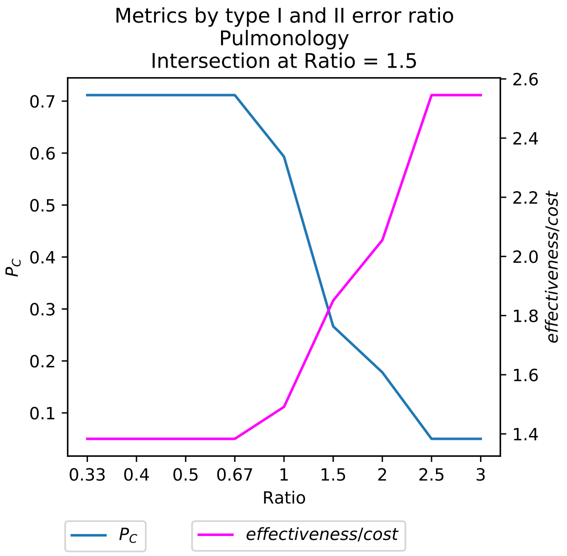

Figure 23: Performance metrics as a function of the type I and II weighting ratio in Pulmonology.

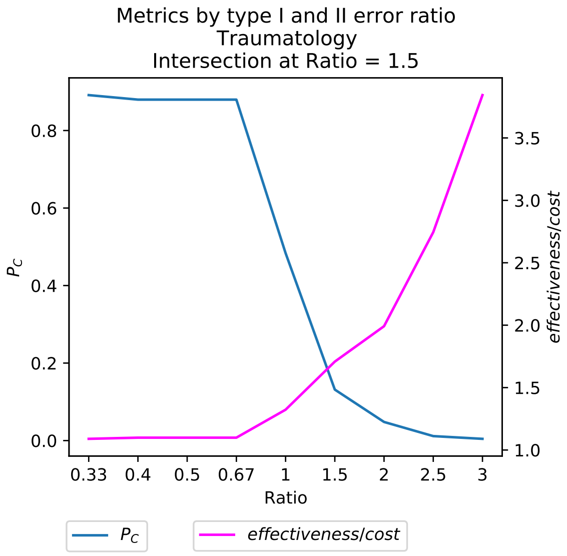

Figure 25: Performance metrics as a function of the type I and II weighting ratio in Traumatology.

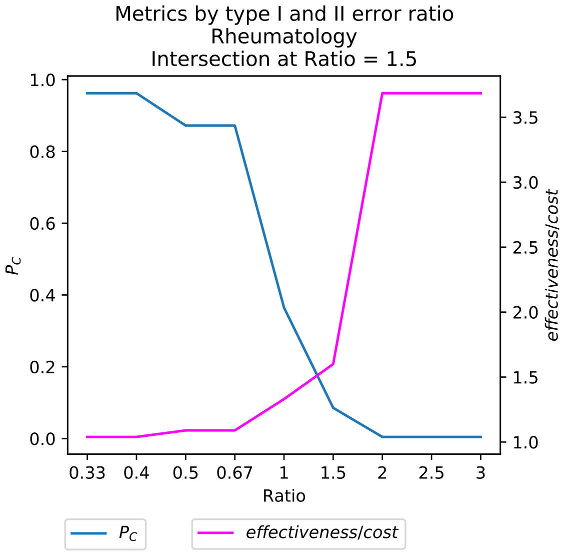

Figure 24: Performance metrics as a function of the type I and II weighting ratio in Rheumatology.

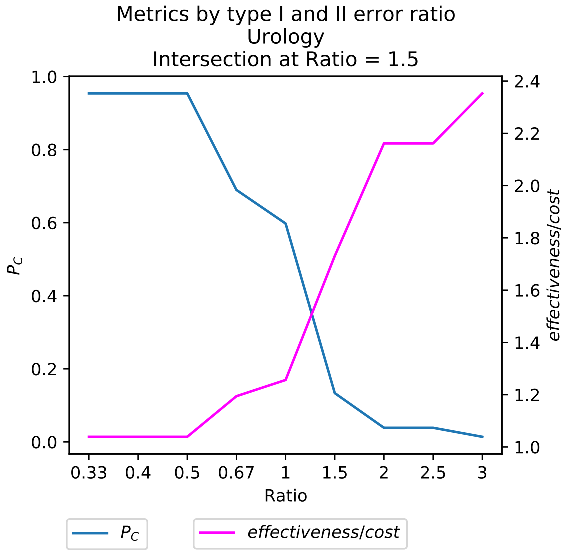

Figure 26: Performance metrics as a function of the type I and II weighting ratio in Urology.
